# Decreased Expression of CPEB3 Predicts a Poor Prognosis in Patients with Melanoma: A Study Based on TCGA Data

**DOI:** 10.1155/2021/8197936

**Published:** 2021-01-11

**Authors:** Xiaomin Zhang, Yanhua Liang

**Affiliations:** ^1^Department of Dermatology, Cosmetology and Venereology, Shenzhen Hospital, Southern Medical University, Shenzhen, Guangdong 518101, China; ^2^The Third School of Clinical Medicine, Southern Medical University, Guangzhou, Guangdong 510515, China; ^3^Department of Dermatology, Shenzhen Hospital (Longgang), Beijing University of Chinese Medicine, Shenzhen 518100, China

## Abstract

**Aim:**

Cytoplasmic polyadenylation element-binding protein 3 (CPEB3) has been acknowledged as a tumor-suppressive gene in several cancers; however, there are few reports on the clinical significance of CPEB3 in melanoma. The aim of this study was to investigate the role of CPEB3 in predicting the prognosis of melanoma patients.

**Methods:**

The association of CPEB3 expression and clinical pathologic features was performed using The Cancer Genome Atlas (TCGA) data set. The role of CPEB3 expression in prognosis was also analyzed. In addition, CPEB3 expression-related pathways were enriched by gene set enrichment analysis (GSEA). Association analysis of CPEB3 gene expression and immune infiltration was performed by ssGSEA.

**Results:**

The mRNA was significantly less in melanoma than in normal tissues (*p* < 0.001). The decrease in CPEB3 expression in melanoma was significantly correlated with T staging (*p* < 0.001), clinical staging (*p* = 0.029), melanoma Clark level (*p* = 0.014), and melanoma ulceration (*p* = 0.003), while it was marginally significant in N staging (*p* = 0.089). Melanoma with low CPEB3 expression was associated with worse OS (overall survival), progression-free survival (PFS), and disease-specific survival (DSS) than in that with high expression. In the univariate analysis, expression of CPEB3, melanoma ulceration, Clark level of melanoma, age, clinical stage, T stage, and N stage were correlated with OS (*p* < 0.05). Further analysis by multivariate Cox regression showed that N stage (*p* = 0.029), melanoma ulceration (*p* = 0.004), and CPEB3 expression (*p* < 0.001) were independent prognostic factors of OS in melanoma. Moreover, GSEA showed that several pathways were enriched in CPEB3, such as PD1 signaling, CTLA4 pathway, CTCF pathway, CHEMOKIN signaling, VEGF signaling, and JAK-STAT pathway. CPEB3 was significantly correlated with the infiltration level of B cells (*p* < 0.001), T cells (*p* < 0.001), T helper cells (*p* < 0.001), and central memory T (Tcm) cells (*p* < 0.001).

**Conclusion:**

CPEB3 may be a potential prognostic marker in melanoma with poor survival. Moreover, PD1 signaling, CTLA4 pathway, CTCF pathway, CHEMOKIN signaling, VEGF signaling, and JAK-STAT pathway may be the key pathway regulated by CPEB3. Moreover, the expression of CPEB3 in melanoma is related to the level of immune infiltration.

## 1. Introduction

Melanoma is a cancer that originates in the melanocytes [1]. Among reported cancers, melanoma is the fifth most common in men and sixth most common in women [2]. Melanoma accounts for about 1% of all skin cancer cases but 70% of skin cancer deaths [2], making it the deadliest form of skin cancer. The 5-year overall survival (OS) rates were 89.6% and 89.1%, respectively, for males and females aged 0–14 years, 84.1% and 93.4% for those aged 15–39 years, and 84.2% and 87.1% for those over 40 years old [3]. In recent years, the incidence of melanoma has been on the rise around the world, with an annual incidence of about 20 per 100,000 [3]. In general, the lifetime risk for melanoma was about 2.4% in Caucasians, 0.5% in Hispanics, and 0.1% in Blacks [3]. It is estimated that by 2020, 100,350 new cases of melanoma will be diagnosed in the US and 6,850 people will die from the disease [4]. The cost of diagnosis and treatment of melanoma is 10 times greater than that of other skin cancer [1]. Ultraviolet (UV) exposure and genetic predisposition remain the most important risk factors for the development of melanoma [1]. Early diagnosis and treatment of melanoma lead to better outcomes, and the introduction of immunotherapy and targeted therapy has provided much needed therapies for advanced disease [2]. However, the percentage of potentially fatal melanoma tumors has remained stable, with very little change in mortality over time [5], which reinforces the importance of continued research on the molecular mechanism of melanoma development and potential diagnostic and therapeutic targets.

Several prognostic biomarkers of melanoma have been reported, including 5-S-cysteinyldopa (5SCD), S100B, and serum lactate dehydrogenase (LDH). Wakamatsu et al. reported that serum 5SCD level is more sensitive than LDH in advanced melanoma [6]. However, the significance of 5SCD and S100B in the serum in the early detection of distant metastases was still controversial [7, 8]. Nonetheless, melanoma is one of the most malignant cancers in the world, and more efforts are needed to identify reliable biomarkers for reliable prediction of survival and recurrence and efficient monitoring of melanoma.

The cytoplasmic polyadenylation element-binding protein (CPEB) family is associated with specific sequences in the untranslated regions of mRNA 3′ to regulate protein translation [9]. Several CPEB-regulated mRNAs regulate cell cycle progression, govern senescence and embryonic cell divisions, and establish cell polarity [9]. CPEB3 is a crucial modulator of cytoplasmic polyadenylation [10] and was downregulated in colorectal cancer (CRC) and ovarian clear cell adenocarcinoma [11]; therefore, it is considered to be a tumor suppressor. Skubal et al. found that expression of CPEB3 was positively correlated with tumor progression and malignancy but negatively correlated with phosphorylation of CPEB3, which may be regarded as a marker for prolonged survival in glioma patients [12]. On the other hand, it has been found that CPEB3 inhibited the proliferation, migration, invasion, and apoptosis of hepatocellular carcinoma (HCC) cells, leading to G0/G1 arrest [11].

As far as we know, the function of CPEB3 in melanoma has not been reported. Here, our current study was aimed at focusing on the association of CPEB3 and prognosis of melanoma. Firstly, we comprehensively analyzed the gene expression matrix, clinical information, and prognosis information of cutaneous melanoma patients based on TCGA database. Secondly, we analyzed the GSEA based on the level of gene expression. In addition, the tissue microenvironment of tumor cells is an important factor of tumors; thus, we also explored the relevance between immune cells and CPEB3 in the immune microenvironment of melanoma. This study suggests that CPEB3 may serve as a therapeutic target and prognostic indicator for cutaneous melanoma.

## 2. Materials and Methods

### 2.1. Collection and Analysis of Data

A total of 472 RNAseq data and corresponding clinical information of patients with melanoma were downloaded from the SKCM project of The Cancer Genome Atlas (TCGA), and those without clinical information were discarded. Finally, a total of 468 RNAseq data with clinical information level 3 HTSeq-FPKM were converted into TPM (transcripts per million reads) format for further analysis. Unavailable or unknown clinical information is considered to be a missing value. According to the expression of CPEB3 in tumor samples, it was divided into low- and high-expression groups. The screening conditions were set as follows: cancer type: other; TCGA skin; gene: CPEB3; analysis type: cancer vs. normal; and critical value settings: *p* value = 0.05, ∣log FC | = 2, and gene rank = generation 10%. The normal skin samples were obtained from the GTEx database.

### 2.2. Gene Set Enrichment Analysis (GSEA)

GSEA [13] is a computational method that is based on the entire gene expression matrix. In this study, GSEA generated an ordered list of all genes according to their correlation with CPEB3 expression, and the gene set permutations were performed 1000 times in this study. The expression profiles of 468 samples were input into GSEA. C2.all.v6.2.symbols.gmt was selected as the reference gene set. The threshold value of GSEA of statistical significance was set as *p* < 0.05 and FDR < 0.25 after correction. The adjusted *p* value and normalized enrichment score (NES) were used to sort the pathways enriched in each phenotype. The ClusterProfiler version 3.11 package [14] was used to analyze the GSEA enrichment and visualization.

### 2.3. Immune Infiltration Analysis

The marker gene of 24 immune cells was extracted from the study of Bindea et al. [15]. Single-sample GSEA (ssGSEA) [16] was used to calculate the level of tumor-infiltrating immune cells based on melanoma mRNA TPM data. The correlation between CPEB3 and these 24 kinds of cells was conducted by Spearman correlation. Figures were generated with ggplot2.

### 2.4. Statistical Analysis

All statistical analyses were performed in R version 3.6.2. The Wilcoxon rank sum test was used for analyzing the difference between CPEB3 expression in normal and tumor tissues. The association of CPEB3 expression and clinical pathologic features was performed by using the Wilcoxon signed-rank test, Kruskal-Wallis test, and logistic regression. The relationship between CPEB3 expression and clinicopathological characteristics was analyzed by using the Fisher exact test or chi-square test. In addition, to evaluate the role of CPEB3 expression in prognosis, we used the Kaplan-Meier method and Cox regression. In Cox regression analysis, variables with *p* < 0.1 in univariate Cox regression were incorporated into multivariate Cox regression and the bilateral *p* < 0.05 was considered to be statistically significantly different.

## 3. Results

### 3.1. Association between CPEB3 Expression and Clinical Characteristics

**Figure 1:**
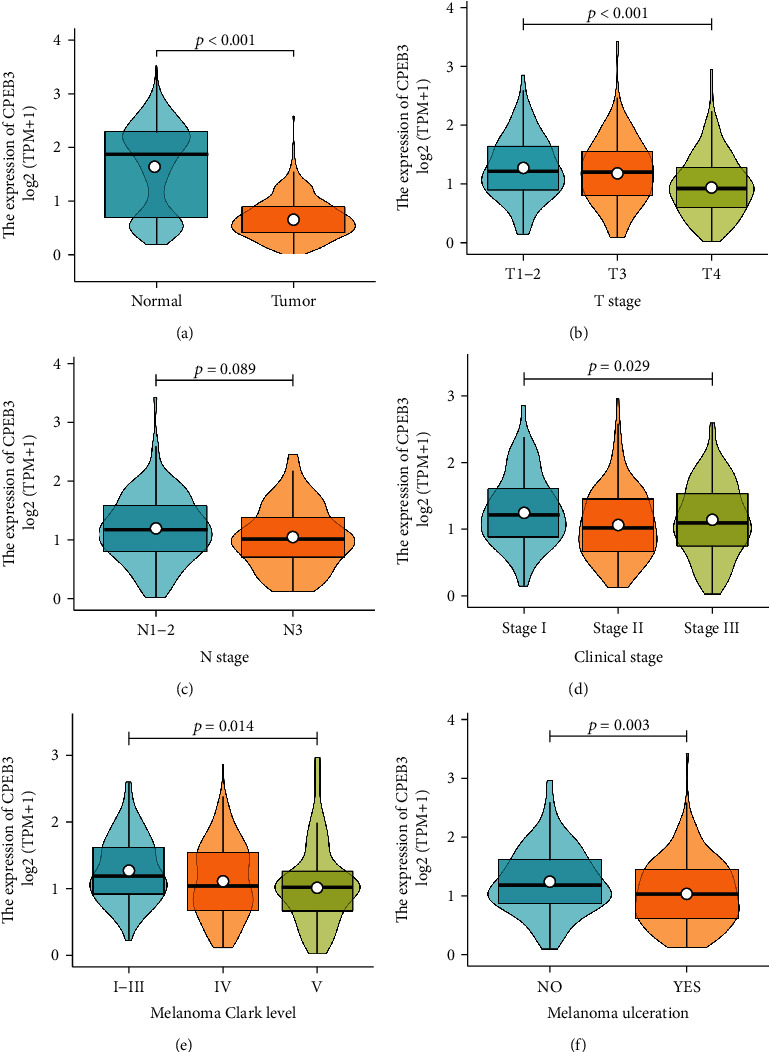
Expression patterns of CPEB3 mRNA in melanoma from 468 RNAseq data. (a) The mRNA was dramatically much less in melanoma than in normal tissues (*p* < 0.001). (b) The most significant difference was from T stage 1-2 to stage 3 and stage 4 tissues (*p* < 0.001). (c) Expression marginally significantly different from N stage 1-2 to stage 3 melanoma in TCGA database (*p* = 0.089). (d) Expression was significantly different from clinical stage I to stage III (*p* = 0.029. (e) Expression reduced gradually from Clark level I-III to level III and IV melanoma. (f) The mRNA was much less in melanoma with ulceration than without ulceration (*p* = 0.003).

The mRNA was much less in melanoma than in normal tissues (*p* < 0.001, Figure 1(a)). The association between CPEB3 expression and clinical characteristics was analyzed by the Kruskal-Wallis test and Wilcoxon signed-rank test: there was a significant correlation between low expression of CPEB3 and higher T staging (*p* < 0.001, Figure 1(b)), clinical staging (*p* = 0.029, Figure 1(d)), melanoma Clark level (*p* = 0.014, Figure 1(e)), and melanoma ulceration (*p* = 0.003, Figure 1(f)), while it was marginally significant in N staging (*p* = 0.089, Figure 1(c)). In addition, similar results were found by using the Fisher exact test or chi-square test (Table 1). Furthermore, univariate logistic regression of CPEB3 expression (Table 2) revealed that CPEB3 expression was also closely related to clinical characteristics, including new event type (metastasis vs. recurrence) (OR = 0.51, 95% confidence interval (CI): 0.26-0.98, *p* = 0.045), T stage (OR = 0.56, 95% confidence interval (CI): 0.37-0.85, *p* = 0.006), melanoma Clark level (OR = 0.54, 95% confidence interval (CI): 0.33-0.87, *p* = 0.012), and melanoma ulceration (OR = 0.51, 95% confidence interval (CI): 0.39-0.95, *p* = 0.029), but not N stage (OR = 0.96, 95% confidence interval (CI): 0.65-1.42, *p* = 0.843), M stage (OR = 1.04, 95% confidence interval (CI): 0.45-2.39, *p* = 0.927), and clinical stage (stage 0-II vs. stage III-IV) (OR = 0.99, 95% confidence interval (CI): 0.68-1.46, *p* = 0.972). These results implied that the expression of CPEB3 affects the initiation and progression of melanoma.

### 3.2. Low CPEB3 Expression Impacts the Prognosis of Melanoma Patients

**Figure 2:**
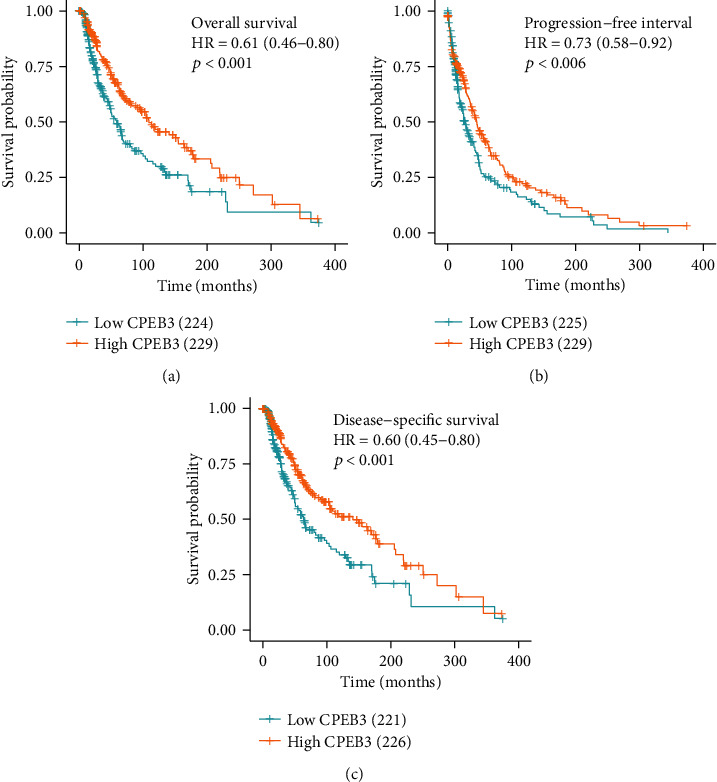
Reduced expression of CPEB3 predicted poorer prognosis in melanoma. Of the 468 cases, patients with low expressions of CPEB3 had significantly shorter overall survival (*p* < 0.001) (a), progression-free interval (*p* = 0.006) (b), and disease-specific survival (*p* < 0.001) (c).

**Figure 3:**
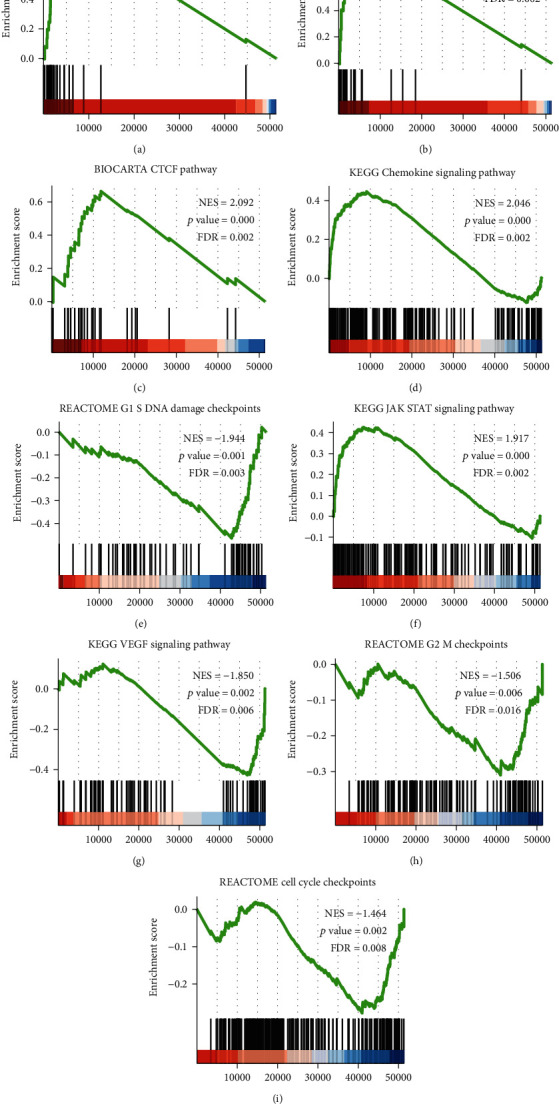
Enrichment plots from gene set enrichment analysis (GSEA). CPEB3 was differentially enriched in PD1 signaling (a), CTLA4 pathway (b), CTCF pathway (c), chemokine signaling pathway (d), G1 S DNA damage checkpoints (e), JAK-STAT signaling pathway (f), VEGF signaling pathway (g), G2 M checkpoints (h), and cell cycle checkpoints (i). ES: enrichment score; NES: normalized ES; FDR: false discovery rate.

As shown in Figures 2(a)–2(c), the OS was significantly worse among patients with low CPEB3 expression than in those with high expression (HR = 0.61, 95% confidence interval (CI): 0.46-0.80, *p* < 0.001, Figure 3(a)). Similarly, the progression-free interval (PFI) (HR = 0.73, 95% confidence interval (CI): 0.58-0.92, *p* = 0.006, Figure 2(b)) and disease-specific survival (DSS) (HR = 0.60, 95% confidence interval (CI): 0.45-0.80, *p* < 0.001, Figure 2(c)) were significantly shorter in the low-expression group than in the high-expression group. In the univariate analysis, the melanoma Clark level, melanoma ulceration, clinical stage, T stage, N stage, age, and expression of CPEB3 affected the prognosis of melanoma patients (all *p* < 0.05). Further analysis by multivariate Cox regression showed that N stage, melanoma ulceration, and CPEB3 expression were independent prognostic risk factors of OS (HR = 0.45 (0.305-0.664), *p* < 0.001, Table 3) in melanoma patients. These results indicated that the level of CPEB3 expression was associated with the prognosis of melanoma patients.

### 3.3. GSEA

Based on the normalized enrichment score (NES), we selected the most significant enrichment signaling pathway with high or low CPEB3 gene expression (Figure 3, Table 4). As shown in Figure 3, GSEA results showed that CPEB3-related melanoma consisted of many key pathways and was associated with tumorigenesis. All of the pathways listed were enriched, including PD1 signaling (Figure 3(a)), CTLA4 pathway (Figure 3(b)), CTCF pathway (Figure 3(c)), chemokine signaling pathway (Figure 3(d)), G1 S DNA damage checkpoints (Figure 3(e)), JAK-STAT signaling pathway (Figure 3(f)), VEGF signaling pathway (Figure 3(g)), G2 M checkpoints (Figure 3(h)), and cell cycle checkpoints (Figure 3(i)).

### 3.4. CPEB3 Expression Is Correlated with Immune Infiltration Level in Melanoma

**Figure 4:**
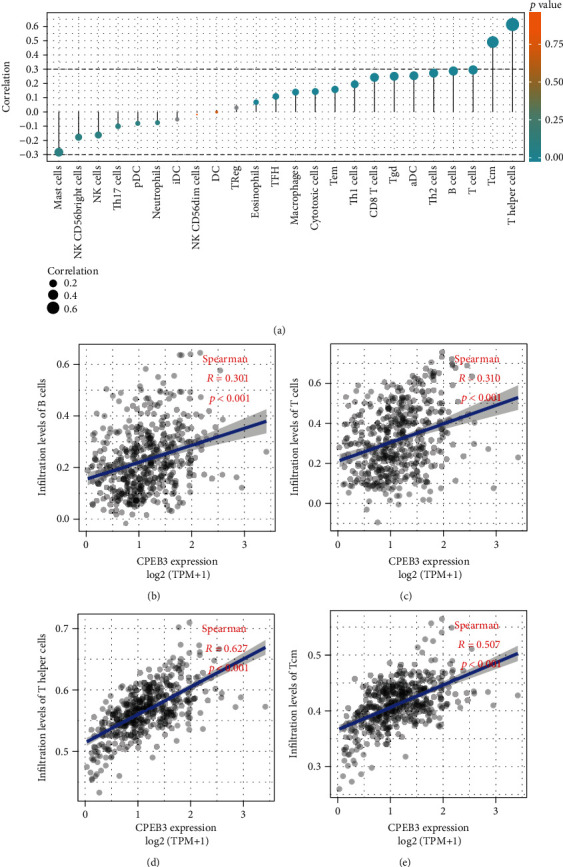
Association analysis of CPEB3 gene expression and immune infiltration: (a) association analysis between CPEB3 expression and immune cells; association analysis of CPEB3 expression with immune infiltration levels of B cells (b), T cells (c), T helper cells (d), and Tcm cells (e).

Tumor-infiltrating lymphocytes are independent predictors of survival in cancers. Therefore, we investigated whether the expression of CPEB3 is related to the level of immune infiltration in melanoma. We evaluated the correlation between CPEB3 and 24 immune cell subsets in melanoma and found that CPEB3 has a close positive correlation with cytotoxic cells, Th1 cells, and CD8 T cells; CPEB3 has a close negative correlation with mast cells, NK cells, and Th17 cells (Figure 4(a)). Further analysis showed that CPEB3 expression was significantly positively correlated with infiltration level of B cells (*R* = 0.301, *p* < 0.001, Figure 4(b)), T cells (*R* = 0.301, *p* < 0.001, Figure 4(c)), T helper cells (*R* = 0.301, *p* < 0.001, Figure 4(d)), and central memory T (Tcm) cells (*R* = 0.301, *p* < 0.001, Figure 4(e)).

## 4. Discussion

Melanoma is one of the few cancers in the United States for which the incidence continues to increase [17]. Recent advances in immunotherapy and targeted therapy have changed the treatment landscape for patients with advanced melanoma in which a small percentage of patients now have extended survivals [18]. However, the mortality of melanoma has remained stable [5], which reinforces the importance of discovering new biomarkers to perform early prediction, diagnosis, prognosis, and therapy of melanoma.

CPEB3 is a member of the CPEB family, which can regulate translation by modulating cytoplasmic polyadenylation [11]. CPEB3 expression is decreased in several tumors. For example, Lin et al. reported that epigenetic silencing of CPEB3 can not only promote the proliferation of CRC cells but also inhibit the apoptosis of CRC cells, providing a new prognostic marker for CRC patients [10]. Similarly, Skubal et al. [12] found that the progression and malignancy of glioma were positively correlated with the expression of CPEB3. Furthermore, Liu et al. [19] indicate that CPEB3 expression levels can be used as a potential biomarker for HCC patient stratification, and more importantly, results from Tang et al. [11] demonstrated that miR-452-3p directly targeted the CPEB3/EGFR axis and accelerated the proliferation and migration of liver cancer cells, which may provide a new therapeutic strategy for the treatment of HCC. Results from our study showed that CPEB3 expression was downregulated in melanoma compared to normal tissues, indicating a potential function for CPEB3 in tumorigenesis. Indeed, higher CPEB3 expression was associated with improved patient survival of melanoma. The OS, DSS, and PFI were significantly worse among patients with low CPEB3 expression than in those with high expression.

We also found that CPEB3 was downregulated in melanoma with a higher clinicopathological grade, such as T staging, clinical staging, melanoma Clark level, and melanoma ulceration. These results indicated that not only can the TNM staging and melanoma Clark level system be used to assess the survival in melanoma but also CPEB3 expression could be used in survival classifiers for melanoma stage evaluation.

To further investigate the role of CPEB3 in melanoma, TCGA data were used for GSEA. GSEA indicated that high CPEB3 expression was hugely enriched in pathways and critical biological functions and was related to tumorigeneses, such as PD1 signaling, CTLA4 pathway, and JAK-STAT signaling pathway. PD1 is a key coinhibitory receptor expressed on T cells after T cell activation. Activation of the PD1 signaling pathway leads to the inhibition of T cell proliferation, activation, cytokine production, metabolic changes, and cytotoxic T lymphocyte killing function, which eventually leads to the death of the activated T cells [20]. CTLA4 is a receptor on the surface of T cells and acts as an immune checkpoint molecule to enhance resistance to homologous antigens [21]. CTLA4-specific or PD1-specific antibodies have produced significant and long-lasting clinical responses in patients with advanced melanoma [22]. The JAK/STAT pathway is involved in cell proliferation, differentiation, migration, and apoptosis, so drugs that act on the JAK/STAT pathway provide an opportunity to treat melanoma [23]. Nevertheless, most patients are still refractory to immunotherapy. Identifying newly predictive biomarkers and designing rational combination therapy become the priorities in melanoma immunotherapy. CPEB3 is an excellent candidate as a potential prognostic marker in melanoma.

Besides, the tissue microenvironment of tumor cells plays a critical role in the development of tumors. In order to conquer some limitations of computational approaches, we evaluated the association between CPEB3 and immune cell populations by screening transcriptomic data. This method could depict the immune infiltrates in tumors. We found a significant correlation between CPEB3 and B cells, T cells, T helper cells, and Tcm cells, and these immune cells are crucial players in cancer control. It is clear that most of T and B cells in the network have a significant positive impact on clinical outcomes. B cells are part of the core immune cell network and are related to prolonging the survival time of CRC patients [15]. Targeting CD4 (+) T helper cells improves the induction of antitumor responses in dendritic cell immunity and improved clinical responses [24]. In addition, Aarntzen et al. demonstrated that expanding Tcm may provide a way to generate a large number of long-lasting antigen-specific cells and enhance the function of T cells in response to melanoma antigens [25]. These results indicate that CPEB3 may be related to the mechanism of immune infiltration and affect the occurrence of melanoma.

Although our analysis in the current study improved our understanding of the relationship between CPEB3 and melanoma, there were still some limitations. First, the results cannot be verified due to a lack of in vitro and in vivo experiments. Second, the data used in our study were accessed from one database, and the results should be cross-validated by multiple data sets. Therefore, some biological information may be omitted in our study.

In summary, low expression of CPEB3 is related to a higher grade and poor prognosis of melanoma. Relatively, high expression of CPEB3 is associated with an increase in immune infiltrating levels in B cells, T cells, T helper cells, and Tcm cells. CPEB3 is correlated with several critical pathways in melanoma, including PD1 signaling, CTLA4 pathway, CTCF pathway, CHEMOKIN signaling, VEGF signaling, and JAK-STAT pathway. Together, these findings indicated that CPEB3 may play a crucial role in immune infiltration and serve as a potential biomarker in the diagnosis and prognosis of melanoma.

## Figures and Tables

**Table 1:** Association of CPEB3 expression and clinicopathological features in melanoma.

Characters	Level	Low expression of CPEB3, *n* (%)	High expression of CPEB3, *n* (%)	*p*
*n*		234	234	
OS event (%)	Alive	118 (51.3)	128 (55.4)	0.429
	Dead	112 (48.7)	103 (44.6)	
T stage (%)	T0	12 (6.0)	11 (6.0)	<0.001
	T1	18 (9.0)	23 (12.5)	
	T2	31 (15.5)	47 (25.5)	
	T3	39 (19.5)	51 (27.7)	
	T4	100 (50.0)	52 (28.3)	
N stage (%)	N0	118 (56.5)	116 (57.4)	0.235
	N1	35 (16.7)	39 (19.3)	
	N2	22 (10.5)	27 (13.4)	
	N3	34 (16.3)	20 (9.9)	
M stage (%)	M0	212 (94.6)	204 (94.4)	1.000
	M1	12 (5.4)	12 (5.6)	
Clinical stage (%)	Stage 0	4 (1.9)	3 (1.5)	0.418
	Stage I	32 (15.0)	44 (21.7)	
	Stage II	78 (36.6)	62 (30.5)	
	Stage III	88 (41.3)	82 (40.4)	
	Stage IV	11 (5.2)	12 (5.9)	
Melanoma Clark level (%)	I	4 (2.5)	2 (1.3)	0.017
	II	7 (4.3)	11 (7.0)	
	III	29 (18.0)	47 (29.7)	
	IV	87 (54.0)	81 (51.3)	
	V	34 (21.1)	17 (10.8)	
Tumor status (%)	Tumor free	102 (44.3)	112 (48.7)	0.400
	With tumor	128 (55.7)	118 (51.3)	
Gender (%)	Female	92 (39.3)	87 (37.2)	0.704
	Male	142 (60.7)	147 (62.8)	
Age (median [IQR])		58.0 [49.0, 71.0]	58.0 [46.0, 71.0]	0.329
Height (median [IQR])		170.0 [164.0, 176.0]	172.0 [162.5, 179.5]	0.304
Weight (median [IQR])		79.0 [67.0, 87.5]	84.0 [70.0, 96.8]	0.012
BMI (median [IQR])		26.0 [23.3, 30.1]	27.5 [24.2, 33.2]	0.026
Tumor tissue site (%)	Extremities	93 (43.5)	102 (50.5)	0.143
	Trunk	95 (44.4)	76 (37.6)	
	Head and neck	22 (10.3)	15 (7.4)	
	Other specify	4 (1.9)	9 (4.5)	
Ulceration (%)	No	65 (40.6)	80 (53.0)	0.039
	Yes	95 (59.4)	71 (47.0)	

IQR: interquartile range. ^∗^Statistically significant.

**Table 2:** Logistic regression analysis of CPEB3 expression associated with clinical pathological characteristics.

Characteristics	Total (*N*)	Odds ratio in CPEB3 expression	*p* value
New event type (metastasis vs. recurrence)	173	0.51 (0.26-0.98)	0.045
T stage (T0-2 vs. T3-4)	384	0.56 (0.37-0.85)	0.006
N stage (N0 vs. N1-3)	411	0.96 (0.65-1.42)	0.843
M stage (M0 vs. M1)	440	1.04 (0.45-2.39)	0.927
Clinical stage (stages 0-II vs. stages III-IV)	416	0.99 (0.68-1.46)	0.972
Melanoma Clark level (I-III vs. IV-V)	319	0.54 (0.33-0.87)	0.012
Tumor status (tumor free vs. with tumor)	460	0.84 (0.58-1.21)	0.35
Tumor tissue site (extremities vs. trunk)	366	0.73 (0.48-1.10)	0.134
Melanoma ulceration (no vs. yes)	311	0.61 (0.39-0.95)	0.029

**Table 3:** Univariate and multivariate Cox proportional hazards analysis for CPEB3 expression.

Characteristics	Univariate analysis		Multivariate analysis	
	Hazard ratio (95% CI)	*p* value	Hazard ratio (95% CI)	*p* value
T stage (T0-2 vs. T3-4)	2.056 (1.502-2.814)	<0.001	1.177 (0.754-1.836)	0.474
N stage (N0 vs. N1-3)	1.711 (1.271-2.304)	<0.001	3.959 (1.152-13.602)	0.029
M stage (M0 vs. M1)	1.734 (0.915-3.287)	0.092	1.507 (0.56-4.056)	0.417
Clinical stage (stage 0-II vs. stage III-IV)	1.579 (1.177-2.118)	0.002	0.546 (0.154-1.935)	0.348
Melanoma Clark level (I-III vs. IV-V)	2.117 (1.472-3.045)	<0.001	1.259 (0.771-2.056)	0.357
Gender (female vs. male)	1.164 (0.872-1.554)	0.301		
Age (≤60 vs. >60)	1.678 (1.266-2.225)	<0.001	1.167 (0.785-1.735)	0.447
Height (>170 vs. ≤170)	0.96 (0.625-1.475)	0.853		
Weight (≤80 vs. >80)	0.853 (0.557-1.307)	0.466		
BMI (≤25 vs. >25)	0.819 (0.508-1.321)	0.414		
Tumor tissue site (extremities vs. trunk)	0.941 (0.693-1.278)	0.698		
Melanoma ulceration (no vs. yes)	2.087 (1.494-2.916)	<0.001	1.793 (1.209-2.66)	0.004
CPEB3 (low vs. high)	0.609 (0.464-0.799)	<0.001	0.45 (0.305-0.664)	<0.001

**Table 4:** Results of gene set enrichment analysis (GSEA).

Description	Set size	Enrichment score	NES	*p* value	*p*.adjust	*q* values	Rank	Leading_edge
REACTOME_PD_1_signaling	23	0.8187355	2.553683	0.0001750700	0.003079053	0.001978739	6472	Tags = 87%, list = 13%, signal = 76%
BIOCARTA_CTLA4_pathway	20	0.8376549	2.518052	0.0001772735	0.003079053	0.001978739	5650	Tags = 80%, list = 11%, signal = 71%
BIOCARTA_CTCF_pathway	24	0.6644226	2.092301	0.0001755926	0.003079053	0.001978739	11813	Tags = 71%, list = 23%, signal = 55%
KEGG_chemokine_signaling_pathway	175	0.4462955	2.046104	0.0001449906	0.003079053	0.001978739	9073	Tags = 39%, list = 18%, signal = 33%
REACTOME_G1_S_DNA_damage_checkpoints	65	-0.4654729	-1.943828	0.0005347594	0.004221891	0.002713178	8556	Tags = 37%, list = 17%, signal = 31%
KEGG_JAK_STAT_signaling_pathway	154	0.4260753	1.916990	0.0001473405	0.003079053	0.001978739	7544	Tags = 31%, list = 15%, signal = 26%
KEGG_VEGF_signaling_pathway	76	-0.4274209	-1.849881	0.0016163793	0.009631181	0.006189433	4420	Tags = 26%, list = 9%, signal = 24%
REACTOME_G2_M_checkpoints	145	-0.3100446	-1.505777	0.0058264336	0.025484595	0.016377554	10391	Tags = 28%, list = 20%, signal = 22%
REACTOME_cell_cycle_checkpoints	263	-0.2780375	-1.464280	0.0022471910	0.012235568	0.007863129	10391	Tags = 25%, list = 20%, signal = 20%

## Data Availability

Data are available upon request.
